# A comparison of thermographic characteristics of the hands and wrists of rheumatoid arthritis patients and healthy controls

**DOI:** 10.1038/s41598-019-53598-0

**Published:** 2019-11-25

**Authors:** Alfred Gatt, Cecilia Mercieca, Andrew Borg, Andrea Grech, Liberato Camilleri, Corene Gatt, Nachiappan Chockalingam, Cynthia Formosa

**Affiliations:** 1 Faculty of Health Sciences, University of Malta, Msida, Malta; 2 Department of Health, University of Malta, Msida, Malta; 3 Faculty of Medicine and Surgery, University of Malta, Msida, Malta; 4 Department of Statistics and Operations Research, Faculty of Science University of Malta, Msida, Malta; 5 Centre for Biomechanics and Rehabilitation Technologies, Staffordshire University, Leek Road, Stoke on Trent, ST4 2DF UK

**Keywords:** Rheumatology, Rheumatoid arthritis

## Abstract

Thermal imaging has been applied to detect possible temperature variations in various rheumatic disorders. This study sought to determine whether rheumatoid arthritis (RA) patients without active synovitis in their hands exhibit different baseline thermographic patterns of the fingers and palms when compared to healthy individuals. Data from 31 RA patients were compared to that of 51 healthy controls. The RA patients were recruited upon confirmed absence of synovitis by clinical examination and musculoskeletal ultrasound. Participants underwent medical infrared imaging of the regions of interest (ROIs). Significant differences were found between the mean temperatures of the palm regions (29.37 °C (SD2.2); n = 306) and fingers (27.16 °C (SD3.2); n = 510) of the healthy participants when compared to the palm regions (31.4(SD1.84)°C; n = 186) and fingers (30.22 °C (SD2.4); n = 299) of their RA counterparts (p = 0.001), with the latter group exhibiting higher temperatures in all ROIs. Logistic regression models confirm that both palm and finger temperature increase significantly in RA without active inflammation. These innovative findings provide evidence that baseline thermal data in RA differs significantly from healthy individuals. Thermal imaging may have the potential to become an adjunct assessment method of disease activity in patients with RA.

## Introduction

Thermography is an emergent technology that has the potential to become an important clinical tool in various fields of medicine, since disease processes can vary the magnitude and pattern of emitted heat in the afflicted person^1^. Besides the obvious effects that vascular perfusion, or sometimes the lack of it, through human tissue may have on body heat emissivity, inflammation also has the potential to alter such heat patterns and magnitude since warmth and localised hyperaemia, are two important components of the inflammatory process^2^.

Persistent synovitis is the hallmark manifestation of RA. Clinical signs of RA include pain and swelling^3^, which often progress to produce deformity unless early aggressive therapy is initiated^4^. Structural damage is associated with irreversible pain and functional impairment^5^, with synovitis being one of the most important predisposing factors preceding structural damage^6^.

In the early stages of the disease, patients may present without apparent joint swelling, making timely detection difficult. Although wider availability of musculoskeletal ultrasound has enabled earlier detection of synovitis, it still has limitations such as access, time required to perform the investigation and being operator dependent^3^. Nonetheless, to date ultrasound has been proven to have the capacity to predict subsequent progression of structural damage following diagnosis of synovitis^7^.

The application of thermography to quantify the effect of disease processes on heat emissivity has been studied in a wide range of conditions ranging from assessment of the diabetic foot to breast cancer^1^. However, evidence of its role in the assessment of inflammation in RA is lacking. Published research to date is characterised by small sample sizes and a lack in methodological consistency. Furthermore, there is conflicting evidence in literature regarding the applicability of thermography in RA. Whilst a study reports that while there may be a role for thermography in assessment of larger joints, it does not appear to be an effective modality for the small joints of the hand^8^, another study reported that the authors demonstrated that infrared can identify the presence of rheumatoid arthritis and the best joints to measure were the metacarpals of the hand, and more specifically the 2nd and 3rd joints^3^. This creates an anomalous situation in this imaging field and, more importantly, currently there is no description of the thermographic characteristics of rheumatoid hands and wrists, which would form the basis on which such studies can be built upon.

The aim of the study was to determine whether patients with established RA but no clinical signs and symptoms of inflammation including pain, swelling and tenderness, would exhibit different thermographic patterns when compared to healthy controls. Consequently this would permit the development of an innovative, noninvasive, inexpensive and more reproducible method to detect the presence of inflammation in RA.

## Results

The RA group consisted of 29 females and 2 males (mean age 60.19years (SD = 12.38); mean height 1.57 m (SD = 0.09); mean weight 75.7 kg (SD = 21.53)). The Healthy group consisted of 51 participants (12 males and 39 females with a mean age of 36years (SD = 12.24), mean weight 70.5 kg (SD = 14) and mean height of 164.5 cm (SD = 9.7)).

Mean duration of RA amongst the study group was 15.2years (SD = 11.9). 29 participants were on Disease Modifying Drugs (DMARDS); out of these, 5 were also on Biologics, 4 were also on glucocorticoids, 2 were also taking NSAIDs, whilst 4 were on DMARDS + glucocorticoids + biologics. 13 were on DMARDS alone. Another participant was on DMARDS + NSAIDs + analgesics. Another participant was on Biologics alone whilst another was on Biologics + Glucocorticoid treatment.

### Univariate statistical tests

No significant differences in mean temperatures were found in the 3 ROIs of the palms of both the healthy and RA group when analysed individually, both for left and right when imaged from the palmar regions; also between the 5 fingers, of both left and right when imaged from palmar regions, of both groups (Table 1).

**Table 1:** ANOVA tests comparing palm regions and fingers of both hands in individual groups.

Regions	Number of regions investigated	p-value
Palms (medial, central and Lateral ROIs) Rt/Lt (Healthy)	306	0.560
Palms (medial, central and Lateral ROIs) Rt/Lt (RA)	186	0.147
Fingers Rt/Lt (Healthy)	510	0.848
Fingers Rt/Lt (RA)	299	0.704

Table 2 however demonstrates significant differences in mean temperatures between the palm and finger regions, for both groups, with the palm being warmer than the fingers(p = 0.001). There were significant differences in both palm and finger mean temperatures between the healthy and RA groups, with the latter group exhibiting higher mean temperatures in both regions. No significant differences in mean temperatures were found between the Left and Right RA wrists. No similar comparison could be carried out with healthy wrists since this data was not available.

**Table 2:** Independent Sample T-test healthy vs RA participants’ different ROIs except for the last test, which tested Left to Right wrists of RA group.

Regions	Number of Regions investigated	Mean Temp °C (SD)	p-value
Palm RA vs Palm Healthy	186 306	31.4(1.84) 29.37(2.2)	0.001
Fingers (RA) vs Fingers (Healthy)	299 510	30.22(2.4) 27.16(3.2)	0.001
Palms vs Fingers (Healthy)	306 510	29.37(2.2) 27.16(3.2)	0.001
Palms vs Fingers (RA)	186 299	31.4(1.84) 30.22(2.4)	0.001
Wrists (RA) Left vs Right	28 27	31.35(0.87) 31.09(0.80)	0.628

### Multivariate statistical tests – logistic regression

In the first Logistic Regression model, the dependent variable is health status (RA, Healthy), while the three predictors are Palm Temperature, Palm location (Medial, Central, Lateral) and Palm Orientation (Left, Right). A Binomial distribution was assumed since the dependent variable has two categories. Table 3 reports potential predictors identified by the logistic regression model for the palm data, whilst Table 4 reports odds ratio and corresponding 95% CI for palm temperature.

**Table 3:** Potential predictors identified by the Logistic Regression model for the palm data.

Likelihood Ratio Tests				
Effect	Model Fitting Criteria	Likelihood Ratio Tests		
	−2 Log Likelihood	Chi-Square	df	P-value
Intercept	543.956	0.000	0	.
Temperature	646.567	102.6	1	0.000
Palm Location	544.361	0.405	2	0.817
Orientation	544.078	0.122	1	0.727

**Table 4:** Odds ratio and corresponding 95% CI for palm temperature.

Effect	B	Std. Error	Wald	P-value	Odds ratio	95% C.I for Odds Ratio	
						Lower Bound	Upper Bound
Intercept	−15.093	1.682	80.472	0.000			
Temperature	0.479	0.055	77.040	0.000	1.614	1.451	1.797

Palm location and Palm Orientation were not found to be significant predictors since the p-values exceed the 0.05 level of significance(p = 0.817 and p = 0.727, respectively). A backward procedure was used to fit the parsimonious model, which identified Temperature as a sole significant predictor (p = 0.0001).

The regression coefficient of Temperature (0.479) is positive indicating that the palm temperature of rheumatoid arthritis participants is expected to be higher than that of healthy participants. The odds ratio (1.614) indicates that for every 1 °C increase in palm temperature, the odds that the participant has rheumatoid arthritis rather than being healthy increases by 61.4%. This odds ratio ranges from 45.1% to 79.7% assuming a 95% confidence level. The Logistic regression model that relates the probability (p) that a participant has rheumatoid arthritis given the palm temperature is:

1 $${\log }_{{\rm{e}}}(\frac{{\rm{p}}}{1-{\rm{p}}})=-\,15.093+0.479\,{\rm{Temperature}}$$

where *p* is the probability that the participant has rheumatoid arthritis and 1- *p* is the probability that the participant is healthy.

**Figure 1:**
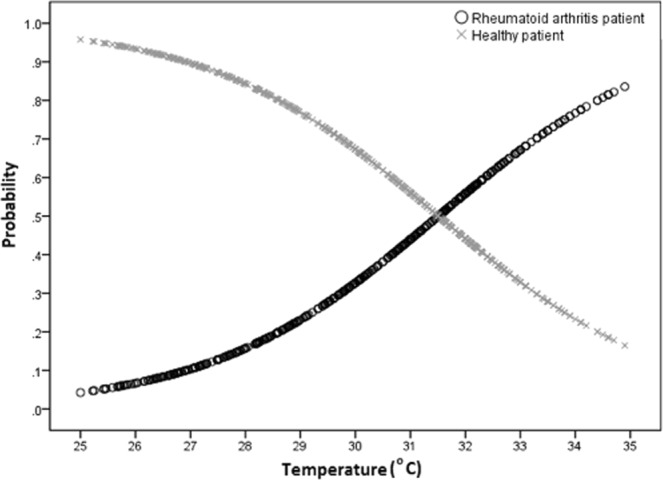
Probability vs temperature curves for palm regions.

The probability curves (Fig. 1) clearly show that the likelihood of rheumatoid arthritis increases as the palm temperature increases.

In the second Logistic Regression model, the dependent variable is health status (Rheumatoid Arthritis, Healthy) while the three predictors are Finger Temperature, Finger location (First, Second, Third, Fourth and Fifth) and Finger Orientation (Left, Right). A Binomial distribution was also assumed since the dependent variable has two categories.

Finger location(p = 0.958) and Finger Orientation (p = 0.836) were not found to be significant predictors. A backward procedure was used to fit the parsimonious model, which identified Finger Temperature as a sole significant predictor (p = 0.001).

Tables 5 and 6 report potential predictors identified by the Logistic Regression model for the finger data and odds ratio and corresponding 95% CI for temperature for finger temperature, respectively.

**Table 5:** Potential predictors identified by the Logistic Regression model for the finger data.

Likelihood Ratio Tests				
Effect	Model Fitting Criteria	Likelihood Ratio Tests		
	−2 Log Likelihood	Chi-Square	df	P-value
Intercept	878.881	0.000	0	.
Temperature	1057.937	179.1	1	0.000
Finger Location	879.527	0.646	4	0.958
Orientation	878.924	0.043	1	0.836

**Table 6:** Odds ratio and corresponding 95% CI for temperature for finger temperature.

Effect	B	Std. Error	Wald	P-value	Odds ratio	95% C.I for Odds Ratio	
						Lower Bound	Upper Bound
Intercept	−10.261	0.844	147.853	0.000			
Temperature	0.338	0.029	138.366	0.000	1.402	1.325	1.483

**Figure 2:**
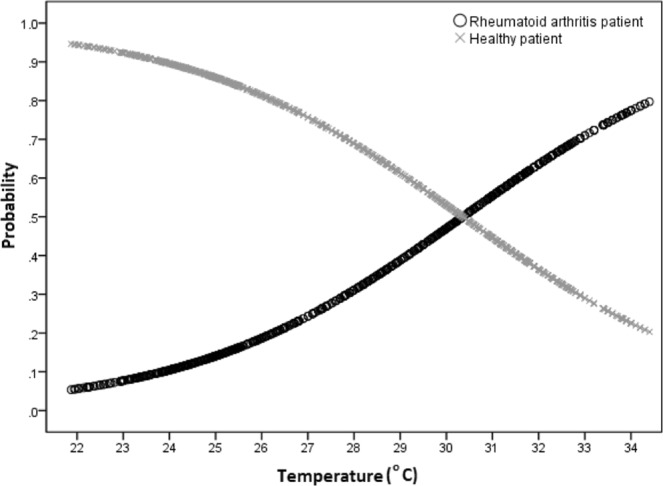
Probability vs temperature curves for finger regions.

The regression coefficient of Temperature (0.338) is positive indicating that the finger temperature of rheumatoid arthritis participants is expected to be higher than that of healthy individuals. The odds ratio (1.402) indicates that for every 1 °C increase in finger temperature, the odds that the patient has rheumatoid arthritis rather than being healthy increases by 40.2%. This odds ratio ranges from 32.5% to 48.3% assuming a 95% confidence level. The Logistic regression model that relates the probability (p) that a patient has rheumatoid arthritis given the finger temperature is:

2 $${\log }_{{\rm{e}}}(\frac{{\rm{p}}}{1-{\rm{p}}})=-\,10.261+0.338\,{\rm{Temperature}}$$

where *p* is the probability that the patient has rheumatoid arthritis and 1- *p* is the probability that the patient is healthy. The probability curves (Fig. 2) clearly show that the likelihood of rheumatoid arthritis increases as the finger temperature increases.

## Discussion

This is the first study exploring thermographic patterns of patients with RA comparing them to healthy controls. Our results have clearly shown that an RA hand without active synovitis exhibits higher temperatures when compared to healthy individuals, with particular thermal properties as outlined in the fitted Logistic regression models.

For palm temperatures, the two probability curves (Fig. 1) intersect at 31.5 °C, which implies that individuals whose palm temperature is less than 31.5 °C are more likely to be healthy; while persons whose palm temperature exceeds 31.5 °C are more likely to have rheumatoid arthritis. Similarly, for finger temperatures, the two probability curves (Fig. 2) intersect at 30.3 °C, which implies that individuals whose temperature is less than 30.3 °C are more likely to be healthy; while those persons whose finger temperature exceeds 30.3 °C are more likely to have rheumatoid arthritis. These innovative findings report for the first time RA thermographic baseline data for the palms, fingers and wrists, making it possible to lay foundations for further studies in this emerging area in clinical practice.

The authors hypothesize that this temperature difference may be attributed to underlying subclinical disease activity or else that the original inflammatory process may cause irreversible thermography changes that persist after the disease activity has resolved. Further studies are required.

Timely detection of ongoing synovitis in RA is of paramount importance to help enable tight disease control. Thermography may be an innovative imaging modality that could detect changes with increased sensitivity compared to other imaging modalities. As a case in point, whilst ultrasonography had not detected any significant changes in our study population, thermography flagged a possible ongoing disease process by reporting these higher temperatures.

Unlike another study in the area^8^, our results clearly demonstrate that there is a significant difference when the images of the palms and fingers of RA patients are compared to those of healthy subjects. The differences in the results could be that Jones *et al*. looked at one point over the MCP joints, whilst in this present study, the temperatures over the whole region of interest, whether finger, palm region, or wrist, were averaged. Additionally, in this cohort, it was ensured that no participant had inflammatory activity going on in any of the regions of interest.

This study demonstrates an important outcome since, before thermography can be employed as a possible clinical tool in the detection of inflammatory disease of the hands, baseline data compared to healthy individuals needed to be established. Once the thermographic patterns of normal and RA hands are known, it may become possible to use this technology to detect abnormal heat signatures in the interphalangeal and metacarpophalangeal joints of the hands.

The applicability of thermography has already been demonstrated in other medical fields such as diabetes, where this modality is becoming more widely accepted as an important diagnostic and screening tool to detect diabetic foot disease^9,10^. Likewise, the authors envisage that this technology will become more widely accepted in the rheumatology field once more robust research is conducted utilizing large sample sizes with random allocations to further confirm the results which this study has reported.

One could argue that one possible limitation of this study is the small sample size. However, the researchers are confident that even if a larger sample size had to be included, results would still remain the same due to the strong statistical results yielded in the study. The same argument could also apply to the fact that not all patients had their absence of synovitis confirmed by ultrasound. However, since all patients presented with a DAS28 score < 2.7 and low CRP level and no joint tenderness or swelling upon the application of the Ritchie Articular Index, the authors are confident that there was indeed no synovitic activities in the concerned joints under investigation.

Another possible limitation of this study is that for each participant the authors measured the temperature only once. Although most previous studies in thermography do not do test-retest as part of their methodology, it would have been ideal if at least a subgroup of participants had been re-tested to confirm data collected, even though a previous study that performed this test-retest on subjects did not find any variability^8^. This is indeed recommended for any future studies involving thermography.

## Method

Thirty-one consecutive RA patients were recruited for this non-experimental comparative study. Ethical approval was sought and obtained from the University of Malta Research Ethics Committee and all participants were treated as per Declaration of Helsinki^11^. All participants provided written informed consent prior to recruitment in the study.

Inclusion criteria were adults aged >18 years, diagnosed with RA by a consultant rheumatologist according to the 2010 Revised American College of Rheumatology and European League Against Rheumatism (ACR/EULAR) Diagnostic criteria^12^. Patients with significant co-morbidities that could possibly affect thermal emissivity of the hands, such as diabetes mellitus, peripheral arterial disease and neuropathy were excluded from the study. RA patients were examined by 2 rheumatologists. Hospital records were consulted to exclude the presence of significant clinical co-morbidities. Joint tenderness, swelling and pain, together with a DAS28 score >2.7, were also exclusion criteria. A random number of participants (n = 21, since for logistical and other personal reasons not all participants could attend) also underwent diagnostic ultrasonography by a trained rheumatologist in order to ensure that the recruited participants had no active signs of synovitis in their hands and wrists^13^.

### Thermography protocol

Thermographic data of RA patients was compared to similar published data of healthy individuals, using the same protocol^14^.

Joint tenderness was assessed by the Ritchie Articular Index (RAI)^15^. As per EULAR criteria, pressure was applied on each joint until there was blanching under the investigator’s fingernail, to apply a consistent amount of pressure. The participants’ responses were then scored with 0 for *no pain*, +1 for *Tender*, +2 for *Tender* and *Winced* and +3 for *Tender, Winced and Withdrew*. If upon the application of the RAI, the participants demonstrated any result >0, the affected joint was excluded from the analyses to ensure that all included joints were pain, tender and swelling-free.

Thermographic images were acquired utilizing a Flir T630 (Flir Systems, Inc., Oregon, USA) thermal camera, following the guidelines of the American Thermology Association^16^ by an experienced thermographer, so that all methods were performed in accordance with the relevant guidelines and regulations in order to ensure both rigor and repeatability^14,16^. The thermal camera employed in this study has an uncooled microbolometer, is precision-made for research and scientific applications, with high resolution, high sensitivity and wide, accurate temperature range featuring a resolution of 640 × 480 pixels, with 307,200 pixels of temperature data in every image captured. Thermal sensitivity/NETD <23 mK@30 °C, a spatial resolution (IFOV) of 0.68mrad, spectral range 7.5 to 14 μm, detector pitch 17 μm.

All participants were instructed not to wear any deodorants, antiperspirants, or other cosmetics that could affect the acquired thermographic pattern on their hands prior to data collection. Participants were acclamatized to the ambient temperature for 20 minutes, as per standard recommendations found in the literature. During this time medical data was collected as outlined above. Participants were also monitored using the thermal camera to confirm that the acclimatization process was taking place^14^.

Thermal images of the palms and dorsal aspect of each hand were recorded. All thermal imaging was performed in the same examination room at a controlled temperature of 23 °C, with the camera mounted on a tripod which was placed at a perpendicular distance of 1.5 m from the subject, who stood in front of a uniform backdrop with hands extended, with fingers and palms spread in front of the cameras during acquisition^14^.

Additionally, normative data of healthy individuals were obtained through a previous study in which two of the researchers (AG, CF) were directly involved during data collection and analysis. This study established normative patterns of the hands and feet in healthy adults^14^. Thus, with appropriate permissions from the concerned research group, raw data (mean temperatures of the regions of interest) were directly available to the researchers. In this previous study, 51 healthy participating adults without a history of significant medical, surgical, vascular or neurological disease were recruited, similarly imaged and analyzed.

### Data extraction and analysis

**Figure 3:**
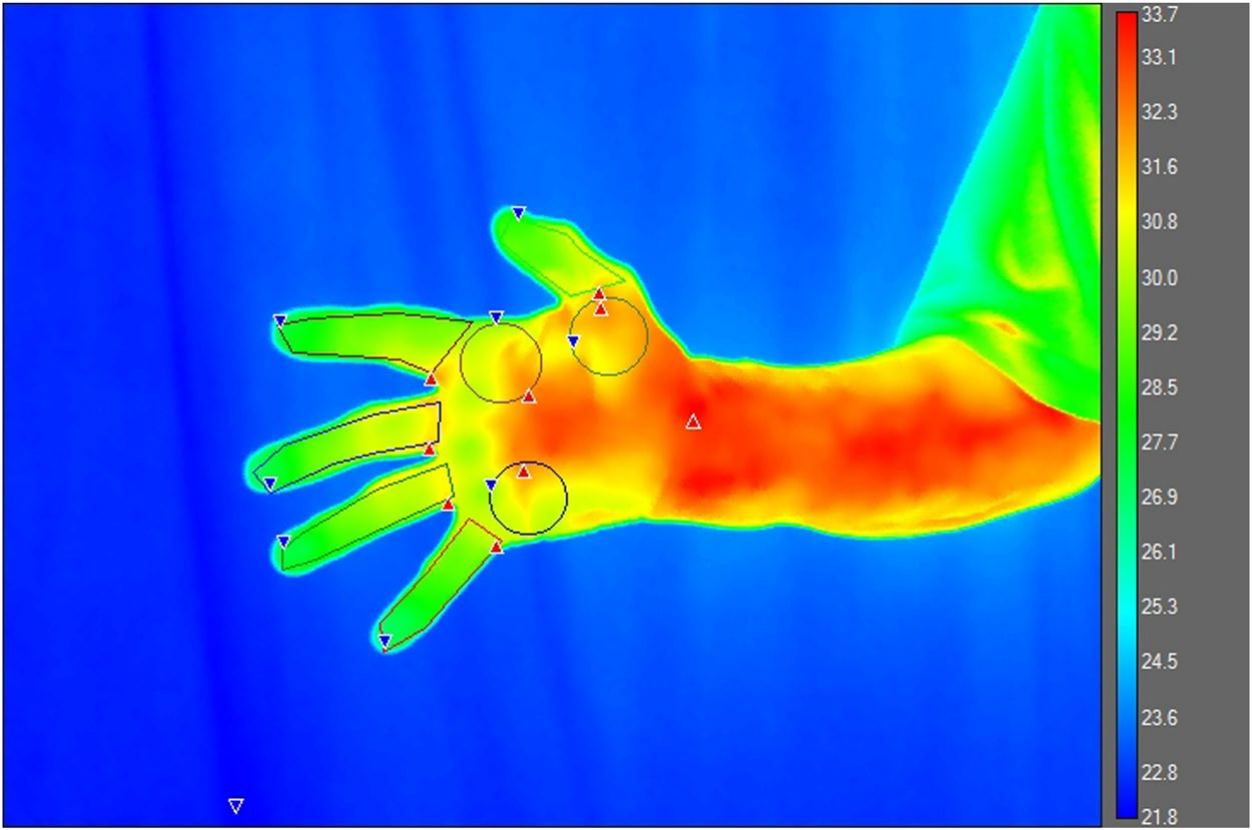
Palm regions of interest.

The thermal images were loaded into FLIR ReseachIR Max software version 4.30.1.70. Regions of interest (ROIs) including the medial, central and lateral palm and fingers from the palmar aspect, were analyzed^14^. Additionally, ROIs for the wrists from the dorsal aspects were obtained (Fig. 3). Those ROIs in which the participant RAI scores ≠0 were excluded from the analysis, as were any ROI’s from which data could not be extracted cleanly due to digital deformities.

The Kolmogorov-Smirnov test was used to assess the temperature distribution of different parts of the palms and fingers, which revealed that all temperature distributions satisfied the normality assumption. As a result, parametric tests were used to compare mean temperatures between the two groups of participants for the same region and between different regions for the same group of participants. The Independent samples t-test was used to compare mean temperatures between two groups or two regions; while the One-Way ANOVA was used to compare mean temperatures between three or more regions.

The major limitation of univariate statistical tests is that they investigate solely the relationship between a dependent variable and a single predictor. It is well known that a single predictor could be rendered a very important contributor in explaining variations in temperatures, but would be rendered unimportant in the presence of other predictors.

To overcome this limitation, statistical modeling and multivariate statistical tests were employed. One of the important contributions in statistical modelling is the concept of generalized linear models (Nelder and Wedderburn, 1972)^17^. For this modelling procedure, Logistic regression analysis was used to relate the logarithm of the odds ratio to the linear combination of 3 predictors (Temperature, Location and Orientation), where the odds ratio represents the odds that a patient with RA compared to the odds that the patient is healthy. Location and Orientation have a nominal scale and Temperature has a metric scale. A separate model was fitted to the palms and fingers data, where both models assumed a Binomial distribution and a Logit link function.

Power analysis for logistic regression models were carried out using the facilities of the Stata program powerlog. To carry out this power analysis, four values are required:

p1: the probability that the response variable equals 1 when the predictor is at the mean

p2: the probability that the response variable equals 1 when the predictor is one standard deviation above the mean

R-squared value which is a measure of goodness of fit

Level of significance (alpha)

Moreover, the size effect size = p2-p1 = 0.26–0.11 = 0.15

**Figure 4:**
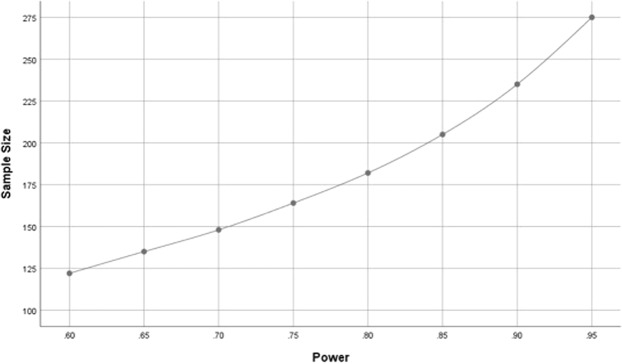
Sample size vs power.

For the palm data, p1 = 0.11, p2 = 0.26, R-squared = 0.42 and level of significance = 0.05. Moreover, the sample comprised 492 (186 + 306) observations. Figure 4 shows that this sample guarantees a power larger than 0.95.

## Data Availability

The authors declare that data will be made available upon request.
